# Supplementary figures and images for: Co-expression of HIF-1α, MDR1 and LAPTM4B in peripheral blood of solid tumors
Source: PeerJ. 2019 Feb 7;7:e6309. doi: 10.7717/peerj.6309 (PMC6368972; doi:10.7717/peerj.6309)

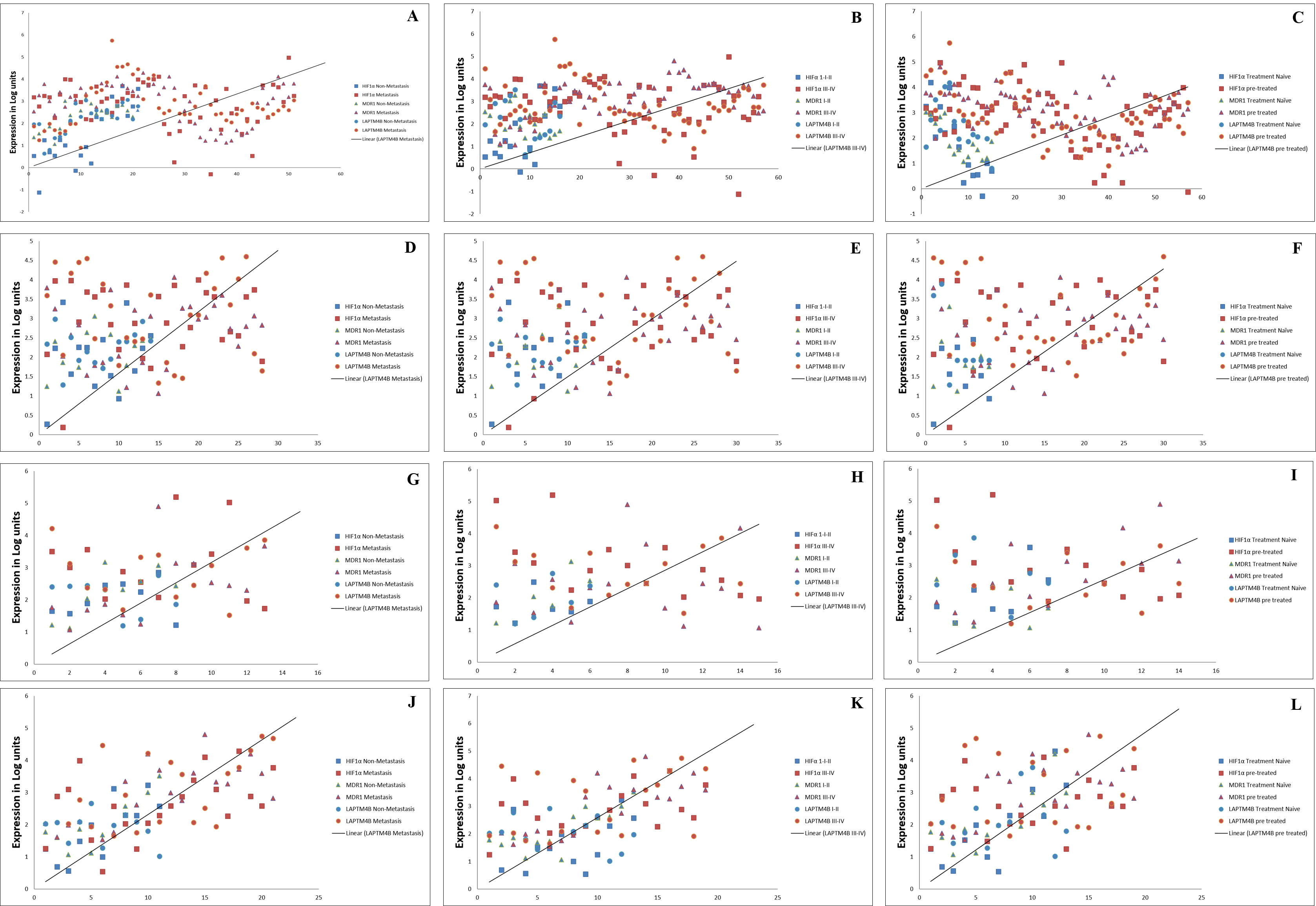

Supplement: Supplemental Information 2 — The scatter plot of expression of three genes in breast cancer with (A) metastasis (B) tumor stage; (C) treatment response. The scatter plot of expression of three genes in ovarian cancer with (D) metastasis (E) tumor stage; (F) treatment response. The scatter plot of expression of three genes in prostate cancer with (G) metastasis (H) tumor stage; (I) treatment response. The scatter plot of expression of three genes in colon cancer with (J) metastasis (K) tumor stage; (L) treatment response. [file peerj-07-6309-s002.png]

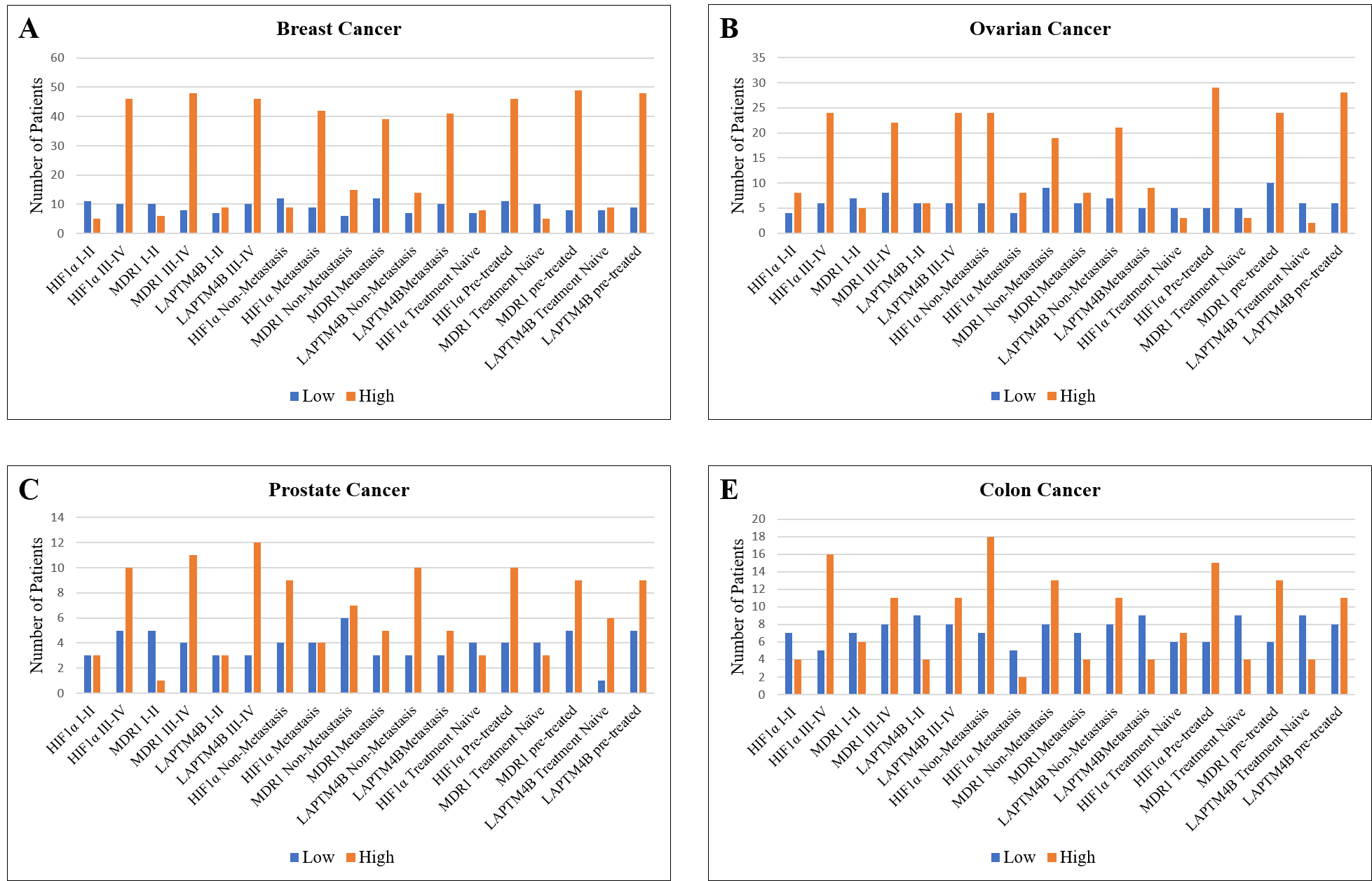

Supplement: Supplemental Information 3 [file peerj-07-6309-s003.png]
